# Prognostic value of triglyceride glucose index in population at high cardiovascular disease risk

**DOI:** 10.1186/s12933-023-01924-2

**Published:** 2023-08-03

**Authors:** Xiao-ling Cai, Yi-fei Xiang, Xiao-fang Chen, Xue-qin Lin, Bi-ting Lin, Geng-yu Zhou, Lin Yu, Yan-song Guo, Kai-yang Lin

**Affiliations:** 1 Cardiology, Department of Cardiology, Fujian Provincial Hospital, Shengli Clinical Medical College of Fujian Medical University, Fuzhou, Fujian Province China; 2 Center for Cardiovascular Epidemiology Research and Prevention Of Fujian Provincial Hospital, Fuzhou, Fujian Province China

**Keywords:** Triglyceride glucose index, High risk cardiovascular disease patients, Prognosis

## Abstract

**Background:**

Early identification of populations at high cardiovascular disease (CVD) risk and improvement of risk factors can significantly decrease the probability of CVD development and improve outcomes. Insulin resistance (IR) is a CVD risk factor. The triglyceride glucose (TyG) index is a simple and reliable index for evaluating IR. However, no clinical studies on the prognostic value of the TyG index in a high risk CVD population have been conducted. This study evaluated the relationship between the TyG index and prognosis in a high risk CVD population.

**Methods:**

This study enrolled 35,455 participants aged 35–75 years who were at high CVD risk and visited selected health centers and community service centers between 2017 and 2021. Their general clinical characteristics and baseline blood biochemical indicators were recorded. The TyG index was calculated as ln[fasting triglyceride (mg/dl)× fasting blood glucose (mg/dl)/2]. The endpoints were all-cause death and cardiovascular death during follow-up. Cox proportional hazard models and restricted cubic spline (RCS) analysis were used to evaluate the correlation between the TyG index and endpoints.

**Results:**

In the overall study population, the mean age of all participants was 57.9 ± 9.6 years, 40.7% were male, and the mean TyG index was 8.9 ± 0.6. All participants were divided into two groups based on the results of the RCS analysis, with a cut-off value of 9.83. There were 551 all-cause deaths and 180 cardiovascular deaths during a median follow-up time of 3.4 years. In the multivariate Cox proportional hazard model, participants with a TyG index ≥ 9.83 had a higher risk of all-cause death (Hazard ratio [HR] 1.86, 95% Confdence intervals [CI] 1.37–2.51, *P*<0.001) and cardiovascular death (HR 2.41, 95%CI 1.47–3.96, *P* = 0.001) than those with a TyG index < 9.83. Subgroup analysis revealed that there was no interaction between the TyG index and variables in all subgroup analyses.

**Conclusions:**

The high TyG index was associated with an increased risk of all-cause death and cardiovascular death in people at high risk of CVD. This finding demonstrates the value of the TyG index in the primary prevention of CVD.

**Trial registration:**

retrospectively registered, the registration number is K2022-01-005 and the date is 2022.01.30.

## Introduction

Cardiovascular disease (CVD) is the leading cause of death worldwide [1]. China has the highest burden of CVD in the world [2]. In 2016, CVD caused nearly 4 million deaths in China, and the incidence of CVD is still increasing [3]. Hypertension, an unhealthy diet, dyslipidemia, diabetes, and air pollution are all significant risk factors for CVD [2]. People with these risk factors have a high CVD burden, an increased incidence of myocardial infarction, unstable angina, heart failure, and other events, a high cardiovascular mortality rate, and a poor prognosis [4, 5]. Early assessment and screening of people at high risk of CVD and intervention for risk factors can prevent at least 80% of CVD events occurrences and have a significant impact on population health [6, 7]. However, there are still few clinical studies on the primary prevention of CVD in high risk groups.

Recent studies have discovered that insulin resistance (IR) is a new independent risk factor for CVD [8], and it can lead to metabolic disorders such as hyperglycemia, hyperlipidemia, and obesity, all of which are common in the high risk CVD population. The triglyceride glucose (TyG) index, combined with fasting blood glucose and fasting blood lipid, is considered as a reliable and simple IR evaluation index. Many previous studies have explored the clinical value of the TyG index in patients with different types of CVD. [9–13] But no studies have been conducted to assess the relationship between the TyG index and prognosis in high risk CVD groups. In order to further investigate the relationship between the TyG index and the primary prevention of CVD risk, in this study we evaluated the relationship between TyG and cardiovascular events in people at high risk of CVD.

## Methods

### Study population

**Fig. 1:**
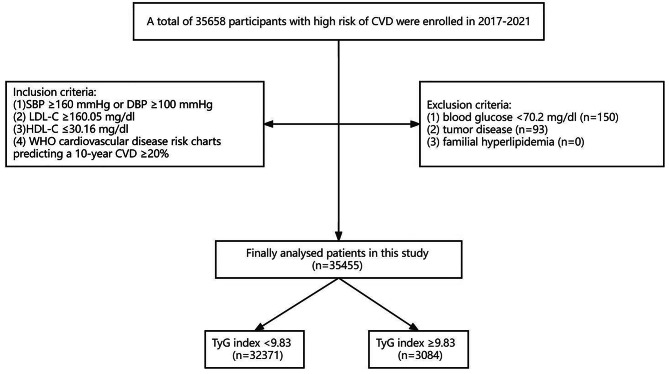
Flow diagram of population selection in this study

This is a large retrospective cohort study that enrolled 35,658 population aged 35–75 years at high risk of CVD from selected health centers and community service centers in nine cities in Fujian Province between 2017 and 2021. Populations at high risk of CVD were defined as having no history of cardiovascular or cerebrovascular disease and meeting one of the following criteria: (1) systolic blood pressure (SBP) ≥ 160 mmHg or diastolic blood pressure (DBP) ≥ 100 mmHg; (2) low-density lipoprotein cholesterol (LDL-C) ≥ 160.05 mg/dl; (3) high-density lipoprotein cholesterol (HDL-C) ≤ 30.16 mg/dl; (4) World Health Organization (WHO) cardiovascular disease risk charts predicting a 10-year cardiovascular disease ≥ 20%. Following the exclusion of 203 people who met one of the following criteria: (1) fasting plasma glucose < 70.2 mg/dl; (2) tumor disease;(3) familial hyperlipidemia, a total of 35,455 people were enrolled in the study **(**Fig. 1**)**. This retrospective study complied with the Declaration of Helsinki and was approved by the Ethics Committee of Fujian Provincial Hospital (Approval No. K2022-01-005).

### Data collection and definitions

Basic information, personal health behaviors, cardiovascular disease history, family history, and other basic data were collected. Fasting venous blood was collected and measured in the laboratory. Total cholesterol (TC), triglycerides (TG), HDL-C, LDL-C, and fasting blood glucose (FBG) were measured.

ln[fasting triglyceride (mg/dl)× fasting blood glucose (mg/dl)/2] was used to calculate the TyG index [14]. Body mass index (BMI) was defined as weight (kg)/[height (m)]^2^. Hypertension was defined as SBP ≥ 140mmHg and/or DBP ≥ 90mmHg in three consecutive measurements on different days without the use of blood pressure medications; or a self-reported history of hypertension. Diabetes mellitus (DM) was defined as random glucose ≥ 11.1mmol/L, or fasting blood glucose ≥ 7.0mmol/L, or 2-hour oral glucose tolerance test (OGTT) glucose ≥ 11.1mmol/L, or a self-reported history of diabetes. Dyslipidemia was defined as TC ≥ 240 mg/dl, LDL-C ≥ 160 mg/dL, HDL-C ≤ 40 mg/dL, or being on lipid-lowering drugs.

### Outcomes

People at high risk of CVD completed questionnaires annually through face-to-face interviews with trained physicians, including records of basic physical exam results, lifestyle factors, medical status, drug use, and routine medical examinations. During the follow-up, the primary outcome was all-cause death. The secondary endpoint was cardiovascular death, which included deaths from diseases with ICD codes I00-I99.

### Statistical analysis

The dose-response relationship between TyG index and all-cause mortality was represented using restricted cubic spline (RCS) analysis. The patients were separated into two groups according to the results of the RCS analysis: the high TyG index group, TyG index ≥ 9.83; and the low TyG index group, TyG index < 9.83. The baseline characteristics of the two groups were presented as mean ± standard deviation (SD) or frequency with percentage as appropriate. The Cox proportional hazards regression model was used to assess the relationship between the TyG index and outcomes, with hazard ratios (HRs) calculated. The findings were presented as HRs with 95% confidence intervals (CIs). Three different models were constructed. Model 1 only included the TyG index. Model 2 adjusted the TyG index, age, and gender. Model 3 adjusted for the variables included in Model 2 as well as diabetes, hypertension, dyslipidemia, smoking, HDL-C, LDL-C, BMI, and SBP. Based on univariate analysis, the Kaplan-Meier survival curves were displayed further. Finally, subgroup analyses were performed, including age (< 60 years versus ≥ 60 years), gender (male versus female), current smoking (yes versus no), diabetes (yes versus no), and BMI (<25 kg/m^2^ versus ≥ 25 kg/m^2^), with the likelihood ratio test used to evaluate interactions between different subgroups. All data were analyzed by R language version 4.2.1, and P < 0.05 was considered statistically significant.

## Results

### Baseline characteristics

**Fig. 2:**
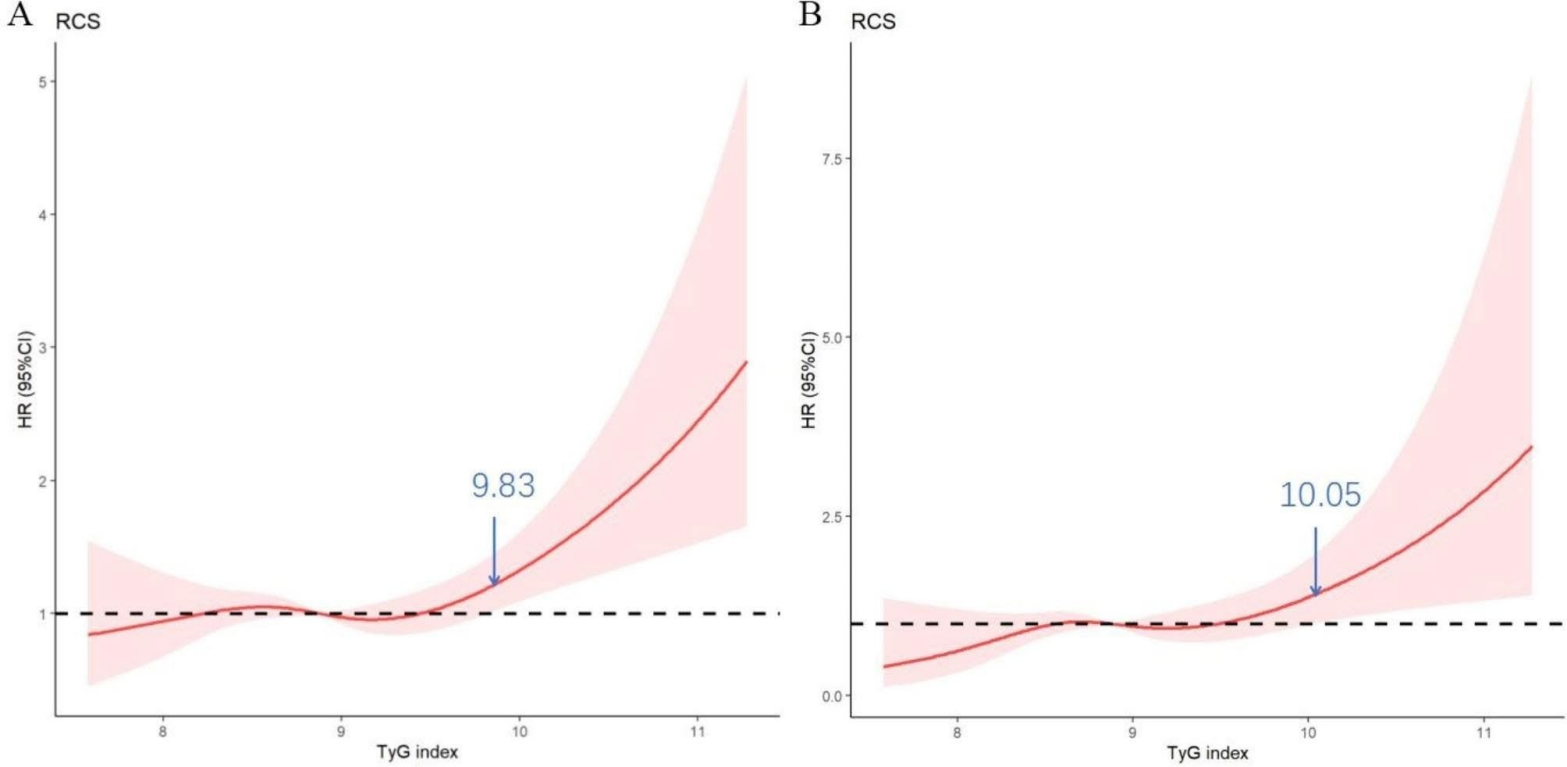
Hazard ratios for A All-cause death and B cardiovascular death based on restricted cubic spines for the TyG index. Red lines represented references for hazard ratios, and red areas represent 95% confdence intervals

A RCS analysis was used to determine whether there was a potential linear or nonlinear association between the TyG index and the risk of all-cause mortality and cardiovascular mortality in a population at high risk of CVD. As can be seen in Fig. 2, the findings of the RCS analysis indicated that the TyG index had a nonlinear connection with both cardiovascular and all-cause mortality. When the TyG index ≥ 9.83, all-cause mortality increases rapidly (HR = 1.20, 95%CI 1.00–1.42, p for non-linearity = 0.031). We divided the patients into two groups using a cut-off value of 9.83 based on the results of the RCS analysis. The baseline characteristics of the 35,455 participants enrolled in this study are shown in Table 1. The overall patients’ mean age was 57.9 ± 9.6 years, 40.7% were male, and the mean baseline TyG index was 8.9 ± 0.6. Patients with a baseline TyG index ≥ 9.83 had lower HDL-C levels and higher levels of TC, TG, FPG, blood pressure, waist circumference, and BMI. They were also more likely to smoke, drink, have diabetes, hypertension, or have dyslipidemia.

**Table 1:** Baseline Clinical Characteristics of participants in different TyG index groups

	Total N = 35,455	TyG index< 9.83 n = 32,371	TyG index ≥ 9.83 n = 3084	P value
Age, years	57.9 ± 9.6	58.0 ± 9.6	57.8 ± 9.5	0.330
Male,n(%)	14,420 (40.7%)	12,907 (39.9%)	1513 (49.1%)	< 0.001
Current smoking,n(%)	7090 (20.0%)	6266 (19.4%)	824 (26.7%)	< 0.001
BMI,kg/m^2^	24.5 ± 3.2	24.4 ± 3.2	25.7 ± 3.1	< 0.001
Waist circumference,cm	84.1 ± 9.2	83.7 ± 9.2	88.1 ± 8.5	< 0.001
SBP,mmHg	146.8 ± 23.4	146.2 ± 23.5	152.9 ± 22.0	< 0.001
DBP,mmHg	85.9 ± 13.2	85.6 ± 13.1	89.4 ± 12.9	< 0.001
Residence:				0.028
Rural	26,053 (73.5%)	23,735 (73.3%)	2318 (75.2%)	
Urban	9402 (26.5%)	8636 (26.7%)	766 (24.8%)	
Laboratory results				
TC,mg/dl	204.90 ± 54.12	204.90 ± 54.12	220.36 ± 61.86	< 0.001
HDL-C, mg/dl	54.12 ± 15.46	54.12 ± 15.46	46.39 ± 15.46	< 0.001
LDL-C, mg/dl	123.71 ± 46.39	123.71 ± 46.39	115.98 ± 54.12	< 0.001
TG, mg/dL	159.1 ± 95.2	138.5 ± 62.7	375.4 ± 107.6	< 0.001
FBG, mg/dL	113.7 ± 33.0	109.5 ± 24.9	157.0 ± 62.9	< 0.001
TyG index	8.9 ± 0.6	8.8 ± 0.5	10.2 ± 0.3	< 0.001
Medical history, n(%)				
Hypertension	24,412 (68.9%)	21,925 (67.7%)	2487 (80.6%)	< 0.001
Diabetes	7966 (22.5%)	6100 (18.8%)	1866 (60.5%)	< 0.001
Dyslipidemia	12,462 (35.1%)	11,265 (34.8%)	1197 (38.8%)	< 0.001
Treatment, n(%)				
Hypotensive drugs	11,649 (32.9%)	10,368 (32.0%)	1281 (41.5%)	< 0.001
Hypoglycemic drugs	3791 (10.7%)	2889 (8.9%)	902 (29.2%)	< 0.001
Lipid-lowering drugs	1668 (4.7%)	1432 (4.4%)	236 (7.7%)	< 0.001
Follow-up time, days	1254.9 ± 463.8	1250.9 ± 464.9	1296.7 ± 449.9	< 0.001

### Association between TyG index and outcomes

There were 551 all-cause deaths and 180 cardiovascular deaths during a median follow-up of 3.4 years. The Cox proportional hazards analysis of the association between the TyG index and all-cause and cardiovascular mortality is shown in Table 2. In univariate analysis, participants with a TyG index ≥ 9.83 had a higher risk of all-cause death (HR 1.56, 95% CI 1.22–1.99, *P* < 0.001) and cardiovascular death (HR 1.85, 95%CI 1.24–2.77, *P* = 0.003) than those with a TyG index < 9.83. After adjusting for potential confounder factors in model 2 and model 3, participants with a TyG index ≥ 9.83 remained at a higher risk of all-cause mortality compared with controls (HR 1.60, 95% CI 1.25–2.05, *P* < 0.001; HR 1.86, 95%CI 1.37–2.51, *P* < 0.001; respectively). Similar results have been observed for cardiovascular mortality (HR 1.93, 95% CI 1.29–2.89, *P* = 0.001; HR 2.41, 95%CI 1.47–3.96, *P* = 0.001; respectively).

**Table 2:** Multivariable Cox regression analyses for the association between TyG index and endpoints

	Model 1		Model 2		Model 3	
	h (95% CI)	*P* Value	HR (95% CI)	*P* Value	HR (95% CI)	*P* Value
All-cause death	1.56 [1.22, 1.99]	< 0.001	1.60 [1.25, 2.05]	< 0.001	1.86 [1.37, 2.51]	<0.001
Cardiovascular death	1.85 [1.24, 2.77]	0.003	1.93 [1.29, 2.89]	0.001	2.41 [1.47, 3.96]	0.001

Model 1: unadjusted

Model 2: adjusted for age and gender

Model 3: adjusted for age, gender, diabetes, hypertension, dyslipidemia, current smoking, HDL-C, LDL-C, BMI, and SBP.

**Fig. 3:**
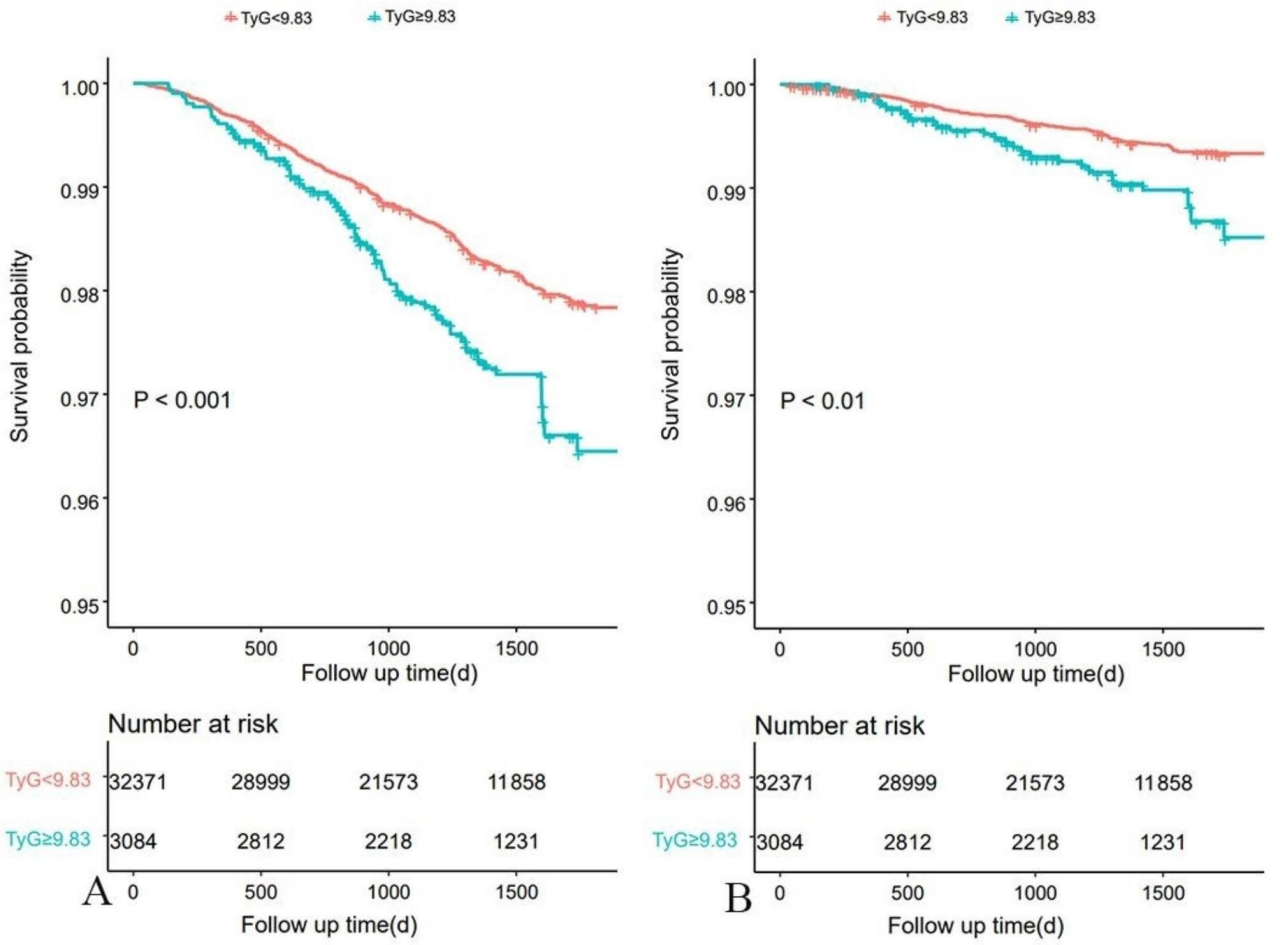
Kaplan–Meier analysis of A All-cause death and B cardiovascular death in various TyG groups

The Kaplan–Meier survival analyses indicated that the TyG index ≥ 9.83 group had a significantly higher cumulative rate of all-cause mortality and cardiovascular mortality than the TyG index <9.83 group. Detailed outcomes of Kaplan–Meier survival analyses are presented in Fig. 3.

### Subgroup analysis

**Fig. 4:**
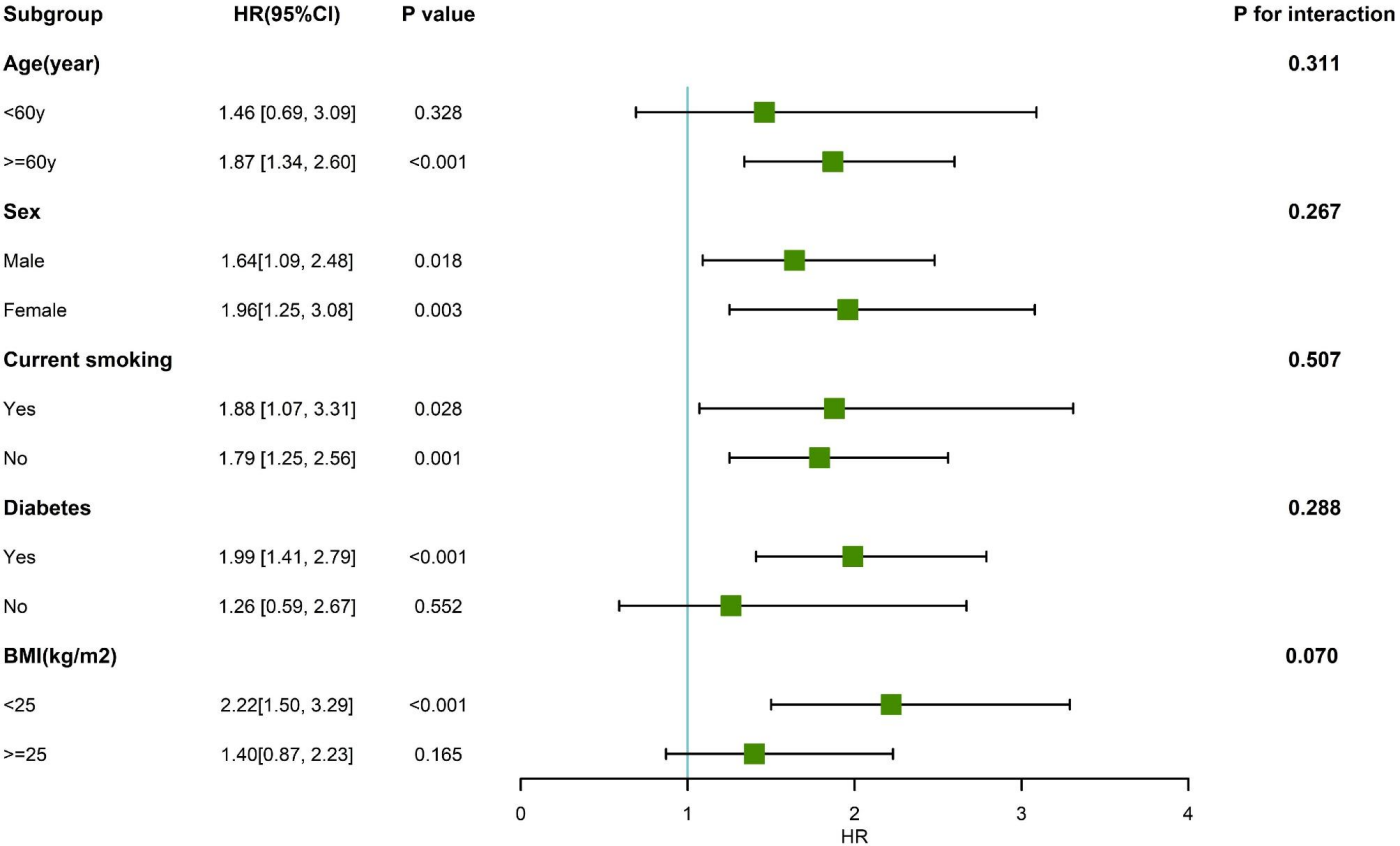
Forest plot of all-cause death according to diferent subgroups. Subgroup analysis included age (< 60 years vs. ≥ 60 years), gender (male vs. female), current smoking status (Yes vs. No), diabetes status (Yes vs. No), and BMI (< 25 kg/m2 vs.≥ 25 kg/m2)

Subgroup analysis and interaction tests were also performed to determine whether there was an interaction between different levels of the TyG index and all-cause mortality in different subgroups. Patients were separated into subgroups based on their age (< 60 years vs. ≥ 60 years), gender (male vs. female), current smoking status (Yes vs. No), diabetes status (Yes vs. No), and BMI (< 25 kg/m^2^ vs.≥ 25 kg/m^2^). As shown in Fig. 4, those who had a TyG index ≥ 9.83 had a higher risk of all-cause death compared to those with a TyG index < 9.83 in subgroups of ≥ 60 years old, history of diabetes, BMI < 25 kg/m^2^, different smoking status, and different genders. In all subgroup analyses, there was no interaction between TyG index and variables (all *P* for interaction > 0.05).

## Discussion

In this retrospective analysis, we verified the association between the TyG index and cardiovascular and all-cause mortality in primary prevention. We discovered that those with a TyG index ≥ 9.83 who had a high risk of CVD also had a higher risk of both cardiovascular and all-cause mortality.

The number of people at high risk of CVD is large, and it is growing year by year as the population ages and unhealthy lifestyles develop [15–17]. A study published in The Lancet Public Health in December 2020 by China’s National Center for Cardiovascular Diseases studied the cardiovascular health and risk factors of 980,000 people on the Chinese mainland from 2015 to 2019. The results showed that 16.6% of people had a high risk of CVD [3]. High risk individuals have a poor prognosis, a high rate of cardiovascular death, and a high prevalence of CVD [4, 5]. Therefore, primary prevention targeting people at high risk of CVD is critical, and early identification, intervention, and improved prognosis will significantly improve community health and lower the burden of illness [18–21].

Insulin resistance is a condition in which the body’s sensitivity and responsiveness to insulin are reduced [22]. IR is an independent risk factor for CVD and can promote metabolic diseases such as obesity, hypertension, hyperlipidemia, and diabetes, and these metabolic disorders are frequently observed in high risk groups for CVD [23]. The TyG index, which combines fasting blood glucose and fasting blood lipid levels, was proposed in 2008 and is regarded as a reliable and simple IR assessment indicator [14]. Existing clinical studies have shown that the TyG index is associated with a poor prognosis of CVD in several groups, including ACS, stable coronary heart disease, unstable coronary heart disease, and so on. [9] In a study of 662 patients over the age of 80 with ACS, Jiao et al. discovered that TyG index was an independent predictor of long-term all-cause mortality (HR: 1.64, 95%CI: 1.06–2.54) and MACE (HR: 1.36, 95%CI: 1.05–1.95) [10]. Gou et al. conducted a retrospective study on 546 patients with CHF and T2DM. Results showed that TyG index was positively correlated with cardiovascular death (HR: 4.42, 95%CI: 1.49–13.15) [11]. Similarly, Liu et al. conducted a prospective study in 1,467 patients with both coronary heart disease and hypertension. The results showed that for every standard deviation (SD) increase in the TyG index, the risk of all-cause death and non-fatal cardiovascular events in this population increased by 28% (HR: 1.28, 95% CI: 1.04–1.59) [24].

In conclusion, the TyG index can be a useful biomarker to identify poor prognosis and assist in further risk stratification of patients with ACS, heart failure, etc. However, current studies on the relationship between the TyG index and CVD mainly focus on secondary prevention, and there are no clinical studies to explore the relationship between the two in primary prevention. And it is unknown if the TyG index predicts poor outcomes in high risk CVD groups. Thus, our observations fill an important knowledge gap in the field. Our study confirms prior findings that the TyG index is a strong predictor of poor outcomes in people at high risk of CVD, even after controlling for any confounding factors. We observed that the TyG index ≥ 9.83 was associated with an elevated risk of all-cause death and cardiovascular death in the high risk CVD population (HR:1.86, 95%CI: 1.37–2.51,P < 0.001; HR:2.41, 95%CI: 1.47–3.96, P = 0.001). In addition, subgroup analysis was conducted, and it was found that the TyG index had a greater ability to predict all-cause mortality in the majority of subgroups. This suggests that the TyG index is a generally stable and reliable prognostic indicator in high risk CVD people, unaffected by gender, smoking, or other factors, and that it can be widely used in high risk CVD people.

It is important to note that the exact cut-off value of the TyG index associated with poor prognosis varied across studies, which we considered to be mainly due to the heterogeneity of the study population. For example, among cancer survivors, patients with a TyG index > 8 had a higher risk of primary cardiovascular events [25]. This may be related to the long-term anticancer treatment of cancer survivors, insufficient attention to and treatment of metabolic abnormalities such as blood lipids and blood glucose. For critically ill patients, a TyG index > 9.2 was significantly associated with poor prognosis, because IR related pathological states were important causes of aggravation in ICU patients [26]. Our study focused on high risk people who have not yet developed CVD, concentrating on five major risk factors that are significantly associated with cardiovascular disease: severe hypertension, diabetes, severe dyslipidemia, gender, and smoking. These risk factors have been confirmed to be closely related to endothelial dysfunction, oxidative stress, and the inflammatory response, and the above-mentioned pathological processes are just mutually reinforcing with the pathological processes of IR. Compared with critically ill patients and tumor survivors, the study included population had a higher TyG index cut-off value, which may be related to the severe metabolic abnormalities and low levels of stress and inflammation in the high risk CVD population included in this study.

Although the cause of the association between TyG index and poor prognosis in the high risk CVD group is yet unknown, the following factors could play a role: First, IR results in an imbalance between the metabolism of glucose and lipids, which in turn promotes inflammation and oxidative stress, causes atherosclerosis to appear and develop, and accelerates the evolution of coronary heart disease [27]. Second, IR can induce increased production of glycosylated products and free radicals, cause an increase in reactive oxygen species (ROS) production, and cause vascular endothelial injury [28, 29]. Third, IR can reduce platelet anti-aggregation sensitivity to prostaglandins I2 and NO, leading to overactivation of platelets, promoting thrombosis and inflammation, causing adverse cardiovascular events and leading to poor prognosis [30]. Fourth, over-glycosylation induced by insulin resistance leads to smooth muscle cell proliferation and collagen deposition, which leads to increased heart stiffness, myocardial fibrosis, and ultimately poor prognosis such as heart failure [9, 22].

The main strength of this article is that it is the first time to explore the relationship between the TyG index and adverse prognosis of cardiovascular events in high risk populations at the level of primary prevention. However, there are also several limitations to this study. First, this study only included Chinese people, and applying it to other ethnic groups with high cardiovascular risk may have different results. But this study included a large number of people, and fully considered the geographical distribution and social and economic development factors, with a certain representativeness. Second, this is a retrospective study, which cannot fully evaluate the causal relationship between the TyG index and poor prognosis. Third, although some confounding factors were adjusted, our research results would still be affected by residual confounding factors. Finally, due to the limited clinical information collected, we did not study the difference between TyG index and other indicators representing IR in predicting the prognosis of high risk people with CVD.

## Conclusion

In conclusion, this study discovered that the TyG index is associated with a poor prognosis in people at high risk of CVD. In high risk CVD population, the TyG index can be utilized as a predictor and risk stratification tool for all-cause and cardiovascular deaths.

## Data Availability

The information and data of the study population were extracted from Hospital Information System. The datasets are not publicly available because the individual privacy of the participants should be protected. Data are however available from the corresponding author on reasonable request.

## Abbreviations

CVD: Cardiovascular disease
IR: Insulin resistance
TyG: Triglyceride glucose
RCS: Restricted cubic spline
HR: Hazard ratio
CIs: Confdence intervals
SBP: Systolic blood preasure
DBP: Diastolic blood preasure
LDL-C: Low-density lipoprotein cholesterol
HDL-C: High-density lipoprotein cholesterol
WHO: World Health Organization
TC: Total cholesterol
TG: Triglyceride
FBG: Fasting blood glucose
BMI: Body mass index
DM: Diabetes mellitus
OGTT: Oral glucose tolerance test
SD: Standard deviation
